# The IGF signaling axis in thyroid cancer: biological complexity and therapeutic challenges

**DOI:** 10.1530/EC-26-0144

**Published:** 2026-05-12

**Authors:** Youyun Peng, Shaojie Xu, Hanning Li, Xingrui Li, Yaying Du

**Affiliations:** Department of Thyroid and Breast Surgery, Tongji Hospital, Tongji Medical College, Huazhong University of Science and Technology, Wuhan, Hubei, People’s Republic of China

**Keywords:** IGF, thyroid cancer, IGF-1R, IGF-2/IR-A axis, RAI-refractory disease, targeted therapy

## Abstract

Dysregulation of the insulin-like growth factor (IGF) axis plays an important role in thyroid cancer progression, dedifferentiation, and therapeutic resistance. While most differentiated thyroid cancers have favorable outcomes, a clinically significant subset develops aggressive behavior or becomes radioiodine (RAI) refractory, for which effective treatments remain limited. Aberrant activation of IGF ligands, IGF-1 receptor (IGF-1R), insulin receptor isoforms (especially IR-A), and IGF-binding proteins (IGFBPs) enhances oncogenic signaling through the PI3K/AKT and MAPK pathways and disrupts differentiation programs essential for iodine handling. Emerging evidence supports an IGF-2/IR-A-dominant autocrine circuit as a feature of aggressive and RAI-refractory disease, highlighting its potential relevance for biomarker-driven patient stratification. However, the clinical translation of IGF-axis targeting in thyroid cancer remains limited, and IGF-1R-directed monotherapies have shown only modest efficacy owing to signaling redundancy, adaptive resistance, metabolic toxicities, and the lack of validated predictive biomarkers for patient selection. Consequently, current translational efforts increasingly emphasize rational combination strategies, targeted delivery platforms, and molecular imaging approaches. This review summarizes key mechanistic and translational insights into IGF signaling in thyroid cancer and discusses how IGF-axis modulation may be integrated into precision oncology strategies for advanced disease.

## Biological architecture of the IGF system

**Figure 1:**
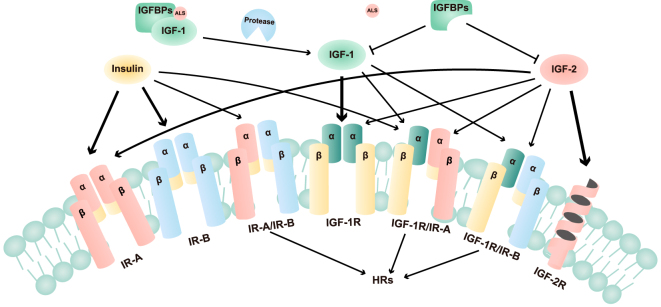
Related molecules, receptors, and the interactions in the IGF system. Diagram illustrating the key ligands (IGF-1, IGF-2, and insulin), receptors (IGF-1R, IGF-2R, and IGF-1R/IR hybrid receptors), IGF-binding proteins (IGFBPs), and their interactions.

The insulin-like growth factor (IGF) system comprises a coordinated network of ligands, receptors, and regulatory proteins. Core components include the peptide ligands IGF-1 and IGF-2, the receptors IGF-1R and IGF-2R, the insulin receptor (IR) isoforms (IR-A and IR-B), and six high-affinity IGF-binding proteins (IGFBPs) (1). Additional regulatory layers involve IGFBP-related proteins, IGFBP proteases, and extracellular matrix (ECM) interactions that fine-tune IGF bioavailability and signaling output (2). An overview of the IGF system architecture is depicted in Fig. 1.

IGF-1 and IGF-2 exert their biological effects primarily through IGF-1R and, albeit with lower affinity, hybrid IGF-1R/IR heterodimers (3). While IGF-1 plays a dominant role in postnatal growth, IGF-2 is particularly active during embryogenesis and is increasingly recognized as a driver of malignant phenotypes in adult tissues (4, 5).

IGF-1R activation triggers autophosphorylation of the intracellular tyrosine kinase domain, initiating downstream PI3K/AKT and MAPK cascades that promote proliferation, survival, and epithelial–mesenchymal transition (EMT) (6, 7). In contrast, IGF-2R lacks intrinsic kinase activity and functions primarily as a scavenger receptor that regulates IGF-2 availability (5). The IR-A, frequently re-expressed in cancer cells, binds IGF-2 with high affinity and activates potent mitogenic signaling (8).

IGFBPs modulate IGF bioactivity primarily through their ability to form binary complexes with IGF ligands and ternary complexes that additionally incorporate the acid-labile subunit (ALS) (9). These complexes differ substantially in stability, circulatory half-life, and tissue distribution. Whereas binary complexes are relatively labile and readily exchange within the extracellular space, ternary complexes serve as the predominant reservoir for circulating IGFs. Their physiological relevance is underscored by ALS-knockout mouse models, in which the absence of ALS disrupts ternary complex formation, markedly reduces circulating IGF and IGFBP3 levels, and results in impaired somatic growth (10). A comparative summary of the structural and functional distinctions between binary and ternary complexes is provided in Table 1.

**Table 1:** Comparative analysis of IGF binary (IGF/IGFBP) and ternary (IGF/IGFBP/ALS) complex characteristics.

Feature	Binary complex	Ternary complex
Composition	IGFs + IGFBPs	IGFs + IGFBPs + ALS
Molecular weight	∼50 kDa	∼150 kDa
Half-life extension	30–90 min (vs 10 min for free IGF)	12–20 h
Transport capacity	Transendothelial transport	Vascular retention
Primary IGFBPs	All IGFBPs	IGFBP3 and IGFBP5
Regulatory mechanism	Spontaneous assembly	GH-dependent ALS synthesis

Beyond ligand sequestration, IGFBPs add an additional layer of complexity to the IGF signaling network by regulating ligand availability, receptor accessibility, and tissue-specific IGF distribution (11). Although historically viewed as inhibitory proteins, certain IGFBPs can enhance IGF signaling by facilitating ligand presentation or stabilizing ligand–receptor interactions (12). Moreover, accumulating evidence demonstrates that IGFBPs also exert important IGF-independent actions, including nuclear translocation and transcriptional regulation, integrin-mediated signaling, and the modulation of DNA damage responses (13, 14). Through these diverse mechanisms, IGFBPs influence cell proliferation, survival, migration, and stress adaptation in a context-dependent manner.

## IGF system in thyroid cancer: mechanisms and clinical relevance

**Figure 2:**
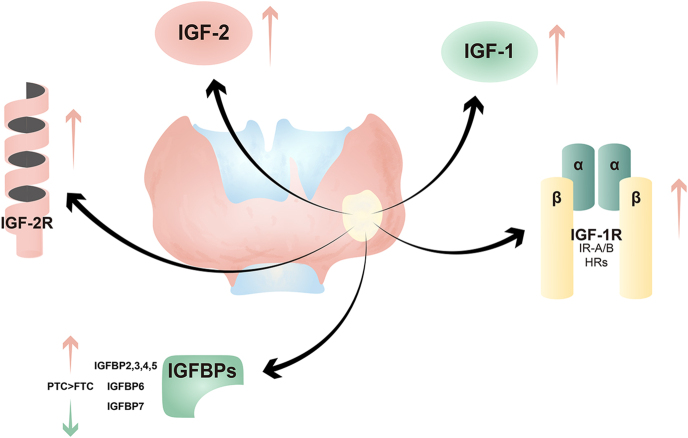
Relationship between the IGF system and thyroid. Increased IGF-1/IGF-2 and receptor expression (IGF-1R, IR isoforms, and hybrid receptors), together with changes in IGFBP expression, are associated with thyroid tumor progression.

Aberrant activation of the IGF axis is increasingly recognized as a key driver of thyroid tumorigenesis and progression. Each major component contributes distinct yet interconnected mechanisms that collectively shape the malignant phenotype of thyroid cancers. Figure 2 illustrates the association between thyroid cancer and the IGF system.

### IGF-1/IGF-1R axis: driver of proliferation and nodule formation

Acromegaly, a chronic condition caused by growth hormone (GH) hypersecretion, is associated with increased cancer incidence and mortality, and thyroid carcinoma represents the most prevalent malignancy in affected individuals (15). This oncogenic predisposition has been mechanistically linked to GH-induced IGF-1 overexpression, suggesting a critical role of IGF-1 in thyroid carcinogenesis (16). Substantiating this association, Tita *et al.* demonstrated that elevated circulating IGF-1 levels promote thyroid cancer initiation and progression through autocrine-paracrine mechanisms (17). Histopathological analysis in 2013 demonstrated significant overexpression of IGF-1 and IGF-1R in thyroid nodules compared to normal thyroid tissues, particularly in follicular adenomas, nodular goiters, papillary carcinomas, and follicular carcinomas (18). This oncogenic process is characterized by a malignancy-dependent gradient in the expression of IGF-1 and its receptor across different thyroid lesions, with levels increasing in the following order: normal tissue < follicular adenoma < nodular goiter < papillary or follicular carcinoma. In addition to ITS malignant potential, a clinical study conducted by Altas *et al.*, involving 80 patients with nodular goiter, demonstrated a positive correlation between the number of nodules and the intranodular levels of IGF-1 and IGFBP3 (19). Beyond proliferation, IGF-1R signaling interfaces with thyroid-stimulating hormone receptor (TSHR) mediated pathways, shifting signaling from differentiation-promoting cyclic adenosine monophosphate (cAMP) responses toward ERK- and AKT-centered cascades (20). This crosstalk may contribute to reduced expression of thyroid-specific genes, impaired sodium/iodide symporter (NIS) function, and susceptibility to radioiodine resistance (7).

### IGF-2/IR-A autocrine loop: a hallmark of aggressive and RAI-refractory disease

Postoperative monitoring of differentiated thyroid cancer (DTC) patients has revealed significant reductions in circulating IGF-2 following surgical resection and radioiodine (RAI) therapy, indicating that tumor tissue may be a primary source of IGF-2 production (21). Consistently, comparative transcriptomic analyses demonstrate that IGF-2 expression is markedly elevated in RAI-refractory tumors relative to RAI-responsive disease, implicating IGF-2 in the development of therapeutic resistance (21, 22). Thyroid carcinomas predominantly express the IR-A, which binds IGF-2 with high affinity and functions as a major mitogenic receptor for IGF-2 in cancer cells (23). The IGF-2/IR-A autocrine loop, thus, establishes a self-reinforcing oncogenic circuit: IGF-2 stimulates IR-A overexpression, and IR-A activation in turn drives persistent mitogenic signaling that promotes tumor progression, aggressive behavior, therapy resistance, and inhibition of apoptosis (21, 24). These findings collectively suggest that therapeutic targeting of IGF-2 or IR-A can substantially reduce thyroid cancer cell proliferation and may provide a promising avenue for treating RAI-refractory disease.

### IGFBPs: context-dependent modulators of thyroid cancer behavior

The six classical IGFBP1–IGFBP6 not only modulate IGF ligand availability but also exert IGF-independent effects that influence thyroid cancer biology. Their functions vary widely depending on cellular context and tumor subtype.

- IGFBP1

While direct evidence linking IGFBP1 to thyroid carcinoma remains lacking, transgenic mouse studies from 1999 demonstrated that IGFBP1 overexpression induced thyroid gland enlargement, suggesting potential compensatory mechanisms within thyroid tissue may counteract IGF-1 bioactivity suppression mediated by IGFBP1 (25).

- IGFBP2

IGFBP2 exhibits significantly elevated mRNA levels in thyroid tumor tissues compared to adjacent normal tissues and may promote the abnormal growth through protecting the IGFs produced by tumor cells (26).

- IGFBP3

IGFBP3 demonstrates a positive correlation between serum levels and nodule dimensions in benign thyroid lesions (19). Lymph node metastasis is a well-recognized critical factor that strongly influences the prognosis of papillary thyroid carcinoma (PTC), and its impact on predicting unfavorable clinical outcomes has been widely emphasized. Consistent with this, a recent bioinformatics analysis further identified IGFBP3 as a potential key regulator, which is not only associated with lymph node metastasis in PTC but also linked to unfavorable prognosis, suggesting its potential role in mediating the prognostic impact of lymph node metastasis (27).

- IGFBP4

Both major subtypes of DTC, follicular and papillary carcinomas, are capable of producing IGFBP4. The secretion of IGFBP-4 is dynamically regulated by signaling cues such as thyroid-stimulating hormone (TSH) and EGF and appears to be closely linked to the differentiation status of tumor cells (28, 29).

- IGFBP5

Upregulated specifically in PTC, IGFBP5 can provide a molecular basis for distinguishing PTC from benign thyroid diseases and thyroid tumors of other molecular subtypes (30).

- IGFBP6

IGFBP6 exhibits differential expression patterns across histological subtypes, showing statistically significant variation between PTC and follicular thyroid carcinoma (FTC), suggesting its potential utility as an auxiliary diagnostic marker for thyroid cancer classification (31).

- IGFBP7

In contrast to the predominantly oncogenic functions observed with other IGFBPs in thyroid carcinoma, IGFBP7 displays a tumor-suppressive profile. The loss of IGFBP7 expression by promoter hypermethylation in PTC has been implicated in tumorigenesis and malignant progression (32).

Collectively, IGFBPs in thyroid cancer do not function as passive IGF carriers, but act as dynamic modulators that integrate differentiation status, metastatic potential, and microenvironmental cues.

### Context-dependent interplay of IGF signaling with canonical thyroid oncogenic drivers, the tumor microenvironment, and redifferentiation strategies

In thyroid cancer, the biological effects of the IGF axis are unlikely to occur in isolation, but rather within the context of canonical driver pathways (7, 33). In particular, in BRAFV600E-driven tumors, IGF signaling may converge with MAPK signaling while simultaneously engaging PI3K/AKT pathways, thereby supporting proliferation, dedifferentiation, and adaptive therapeutic resistance (34). By contrast, in RET-altered tumors, where both MAPK and PI3K/AKT signaling can also be engaged, the IGF axis may function as an accessory amplifier of survival signaling rather than an independent driver. A similar context-dependent interaction may also be relevant in RAS-mutant disease, especially under treatment pressure (33, 34). Thus, in thyroid cancer, the IGF axis may be better viewed as a modulatory signaling node embedded within a broader oncogenic network.

Beyond tumor-intrinsic effects, the IGF axis may also contribute to remodeling of the thyroid tumor microenvironment (TME). In thyroid cancer, direct evidence linking IGF signaling to immune exclusion remains limited. However, growing evidence supports a dynamic thyroid tumor immune microenvironment associated with progression, dedifferentiation, and treatment resistance, while studies in other cancer types have shown that IGF2 can promote fibroblast-mediated immunoevasion (35, 36). These observations support the hypothesis that IGF-related signaling may contribute to stromal adaptation, extracellular matrix remodeling, endothelial interactions, and a more immune-restrictive microenvironment in advanced thyroid cancer, although this requires direct experimental validation (37).

This concept may be particularly relevant in radioiodine-refractory disease, in which dedifferentiation is tightly linked to aberrant MAPK signaling. Because successful redifferentiation therapy generally relies on suppressing MAPK pathway activity, particularly through MEK- or BRAF/MEK-directed strategies, IGF–MAPK/PI3K crosstalk could influence both the magnitude and durability of restored iodine-handling capacity (38, 39). Future studies should, therefore, determine whether IGF-axis status can help identify tumors less likely to respond to redifferentiation alone and whether rational combination strategies may improve restoration of iodine-handling programs.

## Therapeutic targeting of the IGF axis in thyroid cancer

Although the biological rationale for targeting the IGF axis in thyroid cancer is compelling, clinical translation has been challenging. Thyroid cancers are typically driven by canonical oncogenic alterations and marked signaling plasticity, suggesting that the IGF axis may function more often as a cooperative network than as an isolated driver (33, 40). In addition, biomarkers for patient selection remain underdeveloped; although tumor IGF-2 abundance, IR-A predominance, and related receptor-expression patterns are plausible candidate indicators, none has yet been validated for routine clinical stratification (41). These considerations help explain the limited clinical success of IGF-axis-directed therapies in thyroid cancer, while still supporting continued investigation of this pathway (34). Current therapeutic approaches can be broadly categorized into ligand-targeting approaches, receptor-directed therapies, modulation of IGFBPs, natural product-derived agents, and combinatorial targeting strategies.

### Direct IGF ligand targeting

Neutralizing antibodies targeting IGF-1 and IGF-2 suppress ligand-dependent activation of IGF-1R and IR-A while not directly interfering with insulin–IR-B signaling, thereby reducing the risk of metabolic toxicity compared with receptor-targeted strategies (42). In 2011, MEDI-573 was developed as a dual IGF-1/IGF-2-neutralizing antibody that blocks ligand-dependent activation of IGF-1R and IR-A while largely sparing insulin-mediated signaling (43, 44). Given the elevated IGF-2 expression observed in radioiodine-refractory thyroid carcinomas, ligand-directed inhibition may represent a mechanistically plausible approach. BI-836845, a subsequently developed ligand-neutralizing antibody, binds both IGF-1 and IGF-2 with high potency (45). Both agents have demonstrated antitumor activity in preclinical models, particularly in tumors dependent on IGF ligand-driven signaling. Early-phase clinical trials have shown acceptable safety profiles but limited single-agent efficacy, indicating that biomarker-guided patient selection or rational combinations may be required to achieve meaningful clinical benefit. The most common adverse events include hyperglycemia, fatigue, and decreased appetite, typically grade 1–2 in severity. A summary of IGF-targeting mAbs is provided in Table 2.

**Table 2:** Research development of IGF-targeting mAbs.

Therapeutic agent	Molecular target	Indication	Development phase	Reference
Dusigitumab (MEDI-573)	IGF-1/IGF-2	Solid tumors	Phase Ib/II	NCT01498952
Xentuzumab (BI-836845)	IGF-1/IGF-2	Solid tumors	Phase II	EUCTR2013-001110-15-FR
M708.5 (M67)	IGF-1/IGF-2	Solid tumors	Preclinical	(46)
KM-1468	IGF-2	Solid tumors	Preclinical	(47)

### Direct targeting of IGF receptors

Therapeutic strategies targeting the IGF-1R have undergone comprehensive evaluation across preclinical and clinical trial settings, primarily encompassing IGF-1R-directed mAbs and small-molecule TKIs (48).

#### IGF-1R-targeting mAbs

Monoclonal antibodies targeting IGF-1R represented the earliest class of therapeutics directed against this pathway. Although these antibodies show negligible direct affinity for the IR, they can bind and internalize IGF-1R present within IGF-1R/IR heterodimers, potentially affecting downstream metabolic feedback networks. In thyroid carcinoma models, the humanized anti-IGF-1R antibody A12 (cixutumumab) exerts dual effects: *in vitro*, it significantly inhibits the proliferation of anaplastic thyroid carcinoma (ATC) cells directly by suppressing the IGF-1R/AKT signaling pathway, while indirectly impairing the function of tumor-associated endothelial cells (TECs); *in vivo*, it reduces tumor burden and prolongs survival in ATC xenograft models (49). Teprotumumab is the first IGF-1R inhibitor approved by the Food and Drug Administration (FDA) for the treatment of thyroid eye disease (TED), providing clinical proof that IGF-1R antagonism can be therapeutically actionable in thyroid-related disorders. In TED, its efficacy is primarily attributed to modulation of IGF-1R–TSHR crosstalk in orbital fibroblasts rather than direct antiproliferative effects (50). Beyond TED, IGF-1R has been explored as a therapeutic target in several IGF-1R-expressing malignancies, including Ewing’s sarcoma (ES) and hormone receptor-positive breast cancer (BC), although clinical benefits have been limited. Given the frequent upregulation of IGF-1R and its functional interaction with TSHR in thyroid cancer biology, whether IGF-1R-directed therapies can provide clinical benefit in thyroid neoplasms remains an open question that warrants careful, biomarker-driven investigation (51). A summary of IGF-1R-targeting mAbs is provided in Table 3.

**Table 3:** Research development of IGF-1R-targeting mAbs.

Therapeutic agent	Target mechanism	Indication	Development phase	Reference
Teprotumumab (AMG 632/RG-1507)	IGF-1R antagonist	ES, BC	Phase II	NCT00923325 NCT00882674
Cixutumumab (IMC-A12)	IGF-1R antagonist	HCC	Phase II	NCT00831844 NCT00639509
Veligrotug (AVE 1642/VRDN 001)	IGF-1R antagonist	BC, HCC	Phase I	EUCTR2006-005978-51-GB
Lonigutamab (HZ208F2-4)	IGF-1R antagonist/Apoptosis inducer	Solid tumors	Phase I/II	EUCTR2017-001842-82-FR
Ganitumab (AMG-479)	IGF-1R antagonist	Solid tumors	Phase II/III	EUCTR2010-020398-18-HU NCT01024387
Robatumumab (MK-7454)	IGF-1R antagonist	Solid tumors	Phase II	EUCTR2009-011101-16-GB
Figitumumab (CP-751871)	IGF-1R antagonist	NSCLC, ES	Phase II/III	EUCTR2008-004008-30-IE, NCT00560235
Dalotuzumab (MK-0646)	IGF-1R antagonist	Solid tumors	Phase II	NCT01609231
BIIB022	IGF-1R antagonist	Solid tumors	Phase I	NCT00956436
Istiratumab (MM-141)	ERBB3 antagonist/IGF-1R antagonist	Solid tumors	Phase I	NCT01733004

#### IGF-1R TKIs

Small-molecule receptor tyrosine kinase inhibitors (RTKIs) constitute a major class of targeted anticancer therapies (52). As a member of the RTK superfamily, IGF-1R transduces signals through ligand-induced conformational changes that activate its intracellular kinase domain, leading to trans-autophosphorylation of critical tyrosine residues. ATP-competitive RTK inhibitors suppress this process by occupying the catalytic ATP-binding pocket, whereas non-ATP-competitive inhibitors interfere with receptor activation through allosteric or conformational mechanisms, which in certain contexts may enhance kinase selectivity or reduce off-target effects (53).

Since the development of the first IGF-1R-directed TKIs in 2004, multiple candidates have been evaluated in preclinical studies (54). A major obstacle for this therapeutic class is the high degree of structural homology between IGF-1R and the insulin receptor, which predisposes ATP-competitive inhibitors to unintended IR inhibition and consequent metabolic toxicities such as hyperglycemia. Representative IGF-1R TKIs are summarized in Table 4.

**Table 4:** Research development of IGF-1R TKIs.

Therapeutic agent	Mechanism of action	Indication	Development phase	Reference
PL-2258	Small-molecule IGF-1R kinase inhibitor	Solid tumors	Phase I	NCT01779336
Picropodophyllin (AXL-1717/NSC-36407/PPP)	Non-ATP-competitive IGF-1R kinase inhibitor	NSCLC, LUAD	Phase II	NCT01561456
Linsitinib (OSI-906)	ATP-competitive IGF-1R/INSR kinase inhibitor	Solid tumors	Phase II-III	NCT02057380, NCT00924989
BMS-754807	ATP-competitive IGF-1R kinase inhibitor	Solid tumors	Phase I/II	NCT00569036, NCT01225172
KW-2450	IGF-1R/INSR kinase inhibitor	Solid tumors	Phase I	NCT00921336
AQIP	IGF-1R antagonist	Solid tumors	Preclinical	(55)
GSK1904529A	IGF-1R antagonist	Solid tumors	Preclinical	(56)
GTx134	IGF-1R antagonist	Solid tumors	Preclinical	(57)
A-928605	IGF-1R antagonist	Solid tumors	Preclinical	(58)

#### ASOs

Antisense oligonucleotides (ASOs) are chemically modified single-stranded oligonucleotides designed to hybridize with complementary mRNA sequences, leading to either RNase H-mediated target RNA degradation or steric blockade of translation, depending on their chemical design (59). Targeting IGF-1 or IGF-1R with ASOs has demonstrated antitumor effects in preclinical models. In hepatocellular carcinoma, IGF-axis inhibition by ASOs suppresses tumor growth and induces apoptosis, and nanoparticle-assisted delivery has been shown to enhance therapeutic index and reduce systemic toxicity (60). A phase Ib trial (NCT02507583) tested an autologous tumor-cell vaccine incorporating IGF-1R-directed ASOs in patients with glioma. The study showed encouraging signals of prolonged progression-free survival compared with historical controls, although randomized trials are required to confirm clinical benefit (61). Representative IGF-1R-targeting ASOs are summarized in Table 5.

**Table 5:** Research development of IGF-1R ASOs.

Therapeutic agent	Mechanism of action	Indication	Development phase	Reference
ATL-1101	IGF-1R antagonist	PRAD	Preclinical	(62)
IGV-001	IGF-1R modulator/immunostimulant	GBM	Phase II	NCT04485949
CT102	IGF-1R antagonist	HCC	Phase II	CXHL1600204

#### siRNAs

Small interfering RNAs (siRNAs) are double-stranded RNA molecules that are incorporated into the RNA-induced silencing complex (RISC), where the guide strand directs sequence-specific cleavage of IGF-1R mRNA, resulting in efficient posttranscriptional gene silencing (63). In several tumor models, IGF-1R knockdown by siRNAs has been reported to inhibit proliferation and increase sensitivity to chemotherapy, radiotherapy, or targeted therapies (64). A major translational challenge for siRNA-based therapeutics lies in achieving molecular stability, efficient cellular uptake, and tissue-specific delivery. Recent advances in nanomaterial engineering – including lipid nanoparticles and polymer-based carriers – have markedly improved the pharmacokinetic properties and delivery efficiency of siRNA formulations (65).

#### Dominant-negative receptors (DNRs)

DNRs are kinase-deficient IGF-1R variants that heterodimerize with wild-type receptors, generating signaling-incompetent complexes. Early mechanistic studies showed that expression of the IGF-1R 486stop DNR in breast cancer cells impaired β1-integrin-dependent adhesion to laminin and collagen IV while increasing chemosensitivity, suggesting that IGF-1R blockade may influence pathways relevant to metastatic behavior (66). Beyond breast cancer, IGF-1R DNR constructs have also been evaluated in osteosarcoma models, where they upregulated pro-apoptotic genes and inhibited cell proliferation and migration, indicating that dominant-negative inhibition of IGF-1R can suppress tumorigenic phenotypes in multiple sarcoma settings (67). However, because DNRs require gene-based delivery, their current utility remains largely confined to preclinical proof-of-concept studies.

#### Soluble receptor-Fc fusion proteins

To mitigate adverse metabolic effects caused by cross-reactivity between IGF-1R-targeted therapies and the IR due to structural homology, researchers developed IGF-Trap, a soluble fusion protein (68). This molecule consists of the full extracellular domain of IGF-1R fused to the Fc region of human IgG1, demonstrating high-affinity binding to IGFs with negligible insulin binding capacity (three orders of magnitude lower affinity) (68). Subsequent structural optimizations, including linker sequence modifications and hinge region amino acid substitutions, have further enhanced its tumor-targeting specificity and therapeutic efficacy (69).

### Modulation of IGFBPs

Despite the well-established regulatory roles of IGFBPs within the IGF axis, therapeutic manipulation of these proteins in oncology remains largely exploratory. The context-dependent and often bimodal nature of IGFBP function has posed significant challenges for drug development, underscoring the need for deeper mechanistic clarification and rigorous assessment of druggability before IGFBP-directed strategies can realistically progress toward clinical translation.

Nonetheless, several early-phase clinical studies have begun to investigate the feasibility of targeting IGFBPs in human cancers. These efforts include DNA vaccine platforms directed against IGFBP2, as well as antisense oligonucleotides designed to inhibit IGFBP2 or IGFBP5 expression (Table 6). While these agents provide important proof-of-concept evidence supporting IGFBP modulation as a therapeutic approach, all remain in the preliminary stages of development, and none have yet demonstrated efficacy sufficient to justify advancement into late-phase clinical testing.

**Table 6:** Research development of IGFBP targeting therapies.

Therapeutic agent	Type	Mechanism of action	Indication	Development phase	Reference
pUMVC3 (hIGFBP2)	DNA vaccine/therapeutic vaccine	IGFBP2 modulator	OV	Phase I	NCT01322802
AST-201 (EP-201)	DNA vaccine/therapeutic vaccine	IGFBP2 inhibitor/immunostimulant	OV	Phase I	NCT06687941
AST-302 (WOKVAC)	DNA vaccine/therapeutic vaccine	HER2 antagonist/IGF-1R antagonist/IGFBP2 inhibitor	BC	Phase II	NCT04329065
OGX-225	ASO	IGFBP2/IGFBP5 inhibitor	BC	Preclinical	(70)

Taken together, current evidence positions IGFBP modulation not as a validated therapeutic modality but as an emerging conceptual framework that may, with further mechanistic refinement and improved targeting technologies, contribute to future intervention strategies within the IGF signaling network.

### Natural product-derived agents

Several natural compounds have been reported to influence components of the IGF signaling network as part of their broader pleiotropic effects. Resveratrol has been shown to modulate IGF-1/IGFBP3 expression patterns in certain tumor models, although its effects appear highly dose-dependent (71, 72). Curcumin may attenuate IGF-1R-associated signaling among multiple pathways implicated in tumor progression (73). Quercetin has been reported to inhibit IGF-1R activation and partially reverse EMT phenotypes in triple-negative breast cancer cells, but these observations are limited to preclinical studies (74).

Overall, while these natural products exhibit diverse anticancer activities, their IGF-related effects are indirect and context-dependent, and none currently represent validated IGF-targeted therapeutic agents. Further, mechanistic and pharmacologic investigation is required to determine whether modulation of the IGF axis contributes meaningfully to their antitumor activity or potential translational value.

### Combinatorial targeting strategies

**Figure 3:**
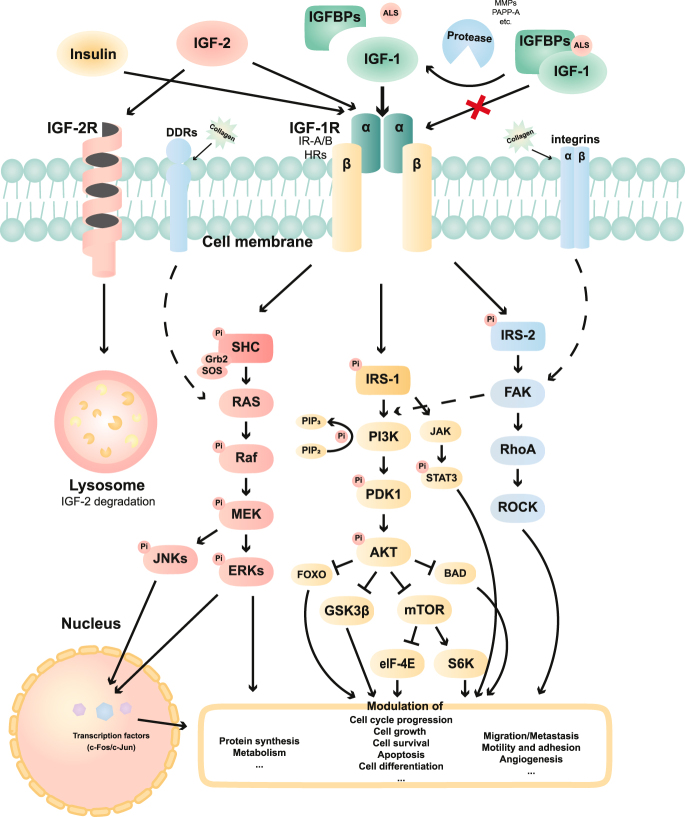
Downstream signaling pathway of the IGF system. Schematic overview of IGF-1, IGF-2, and insulin binding to IGF-1R, IR isoforms, hybrid receptors, and IGF-2R. IGF-2R mediates lysosomal degradation of IGF-2, while IGFBPs and proteases regulate ligand bioavailability. Receptor activation triggers IRS/SHC-dependent signaling (PI3K/AKT/mTOR, MAPK/ERK, JAK/STAT, and FAK/RhoA/ROCK), regulating growth, survival, and migration.

Despite intensive preclinical and early clinical efforts, translation of IGF-targeted therapies has been hampered by the intrinsic complexity of the IGF axis and its extensive crosstalk with metabolic and growth-factor signaling networks. Two major limitations have emerged. First, on-target toxicities arise because key components of the IGF pathway overlap with insulin signaling, and IGF-1R/IGF ligand inhibition can reduce insulin sensitivity or perturb tissue homeostasis. Second, adaptive resistance frequently develops through compensatory activation of parallel pathways or alternative receptor isoforms, a challenge that may be especially relevant in pathway-redundant settings such as thyroid cancer. Consistent with this complexity, IGF-1R activation regulates multiple oncogenic programs, most notably proliferation, survival, EMT, metastatic competence, and therapeutic resistance, primarily via PI3K/AKT and MAPK cascades, with context-dependent engagement of JAK/STAT or FAK signaling (75). The downstream signaling pathways are illustrated in Fig. 3.

This extensive signaling redundancy provides a mechanistic basis for the limited efficacy of IGF-directed monotherapies. Consequently, combinatorial strategies that simultaneously inhibit compensatory upstream receptors or shared downstream effectors have attracted increasing interest as a rational approach to overcoming resistance.

The dual-inhibition strategy achieves three critical objectives: i) pharmacodynamic synergy enabling dose optimization while preserving antitumor efficacy, ii) prevention of adaptive resistance through parallel pathway interdiction, and iii) enhanced therapeutic precision that minimizes off-target tissue toxicity. Table 7 systematically categorizes clinically validated combination regimens demonstrating significant efficacy enhancements in phase I/II trials.

**Table 7:** Research development of combinatorial therapies.

Combination type	Agent	IGF-targeted drug	Indication	Development phase	Reference
mTOR inhibitor	Everolimus	Figitumumab	Tumors	Phase I	NCT00927966
EGFR inhibitor	Erlotinib	Dalotuzumab (MK-0646)	Tumors	Phase I/II	NCT00769483
MEK 1/2 inhibitor	Selumetinib	Cixutumumab	Tumors	Phase I	NCT01061749
CDK4/6 inhibitor	Palbociclib	Ganitumab	ES	Phase II	NCT04129151
SRC inhibitor	Dasatinib	Ganitumab	Rhabdomyosarcoma	Phase I	NCT03041701
AKT inhibitor	MK-2206	Dalotuzumab	Tumors	Phase I	NCT01243762

In addition to combination regimens involving multiple targeted agents, dual-targeting drugs have emerged as an approach to modulate multiple pathways within a single pharmacologic entity. By simultaneously engaging distinct molecular targets, these agents may mitigate certain forms of compensatory signaling that undermine single-agent therapies. Several dual-target inhibitors currently under preclinical or early clinical investigation are summarized in Table 8. Nonetheless, their therapeutic index and true capacity to delay resistance remain to be defined, as most candidates have yet to demonstrate meaningful efficacy in late-phase clinical trials.

**Table 8:** Research development of dual-targeting therapies.

Therapeutic agent	Type	Target(s)	Indication	Development phase	Reference
NVP-TAE226	Small-molecule inhibitor	IGF-1R/FAK	GBM	Preclinical	(76)
SY-707	Small-molecule inhibitor	IGF-1R/FAK/ALK	BC	Preclinical	(77)
LL-28	Small-molecule inhibitor	IGF-1R/SRC	Tumors	Preclinical	(78)
AZD-3463	Small-molecule inhibitor	IGF-1R/ALK	BC	Preclinical	(79)
EI-04	Bispecific antibody	IGF-1R/EGFR	Tumors	Preclinical	(80)

A recent review summarized emerging progress in the development of affibody-based radiometal pharmaceuticals, including exploratory IGF-1R-targeted radioligands with potential applications in early tumor detection, patient stratification, and treatment-response assessment (81). The radiolabeling properties and biological characteristics of representative IGF-1R affibody conjugates are summarized in Table 9.

**Table 9:** Summary of IGF-1R-targeted radiolabeled affibody constructs for tumor theranostics.

Tracer name	Radionuclide	Principle/mechanism of action	Advantages
68Ga-NODAGA-ZIGF-1R:4:40	Gallium-68 (68Ga)	ZIGF-1R:4:40 conjugated with NODAGA and labeled with ^68^Ga for PET	Chelate metals covalently with a minimal impact on ZIGF-1R structure and IGF-1R binding
64Cu-NOTA-ZIGF-1R:4:40	Copper-64 (64Cu)	ZIGF-1R:4:40 linked to NOTA chelator and labeled with ^64^Cu for PET	
111In-DOTA-ZIGF-1R:4551	Indium-111 (111In)	ZIGF-1R:4551 coupled with DOTA for ^111^In radiolabeling for SPECT	
99mTc-ZIGF-1R:4551-GGGC	Technetium-99m (99mTc)	GGGC peptide enables direct labeling of ZIGF-1R:4551 with ^99^ᵐTc for SPECT	Enable direct metal binding via terminal peptide motifs, removing the need for external chelators
[99mTc(CO)3]^+^-(HE)_3_-ZIGF-1R:4551	Technetium-99m (99mTc)	(HE)_3_-modified ZIGF-1R binds [^99^ᵐTc(CO)_3_]^+^ for SPECT	

These advances suggest emerging opportunities for integrating IGF-1R-targeted molecular imaging into precision oncology, particularly for investigational applications such as identifying patients who may benefit from IGF-axis-modulating strategies and exploring imaging-based assessment of treatment response.

## Challenges and future directions

Despite compelling biological evidence implicating the IGF axis in thyroid cancer progression, clinical translation remains challenging (82). To date, no clinical study focused specifically on thyroid cancer has demonstrated a clear therapeutic benefit of IGF-axis inhibition, and the modest activity of first-generation IGF-1R-directed approaches likely reflects several disease-specific challenges. These include signaling redundancy between IGF-1R and IR-A, compensatory activation of MAPK and PI3K/AKT pathways, and the broader context in which many thyroid tumors are primarily driven by canonical oncogenic alterations (40, 75). In this setting, the IGF axis is more likely to function as a context-dependent signaling partner than as a dominant standalone dependency. In parallel, clinically actionable biomarkers remain insufficiently developed; although tumor IGF-2 abundance, IR-A predominance, and related receptor expression patterns are biologically plausible candidate indicators, validated thresholds for routine patient stratification are still lacking. Collectively, these limitations suggest that IGF-targeted monotherapies are unlikely to provide durable benefit in unselected thyroid cancer populations.

Technological advances in molecular imaging and targeted drug delivery may help address some of these limitations. Radiolabeled IGF-1R affibodies represent an emerging noninvasive approach with potential applications in the detection of IGF-activated tumors and refining investigational patient stratification. Nanoparticle-assisted delivery of antisense oligonucleotides or siRNAs targeting IGF-1R or IGFBPs may enhance tumor selectivity, although further pharmacologic and clinical validation is required. Moreover, rational combination regimens may provide a more practical translational direction by counteracting compensatory signaling and improving the durability of antitumor responses.

Future studies should integrate multi-omics profiling, single-cell analyses, and spatial approaches to better define IGF-axis dependencies within the thyroid TME. Particular attention should be paid to how IGF signaling intersects with dedifferentiation, stromal adaptation, immune-restrictive microenvironmental states, and responses to redifferentiation-oriented therapy. In this context, biomarker-driven clinical trials and thyroid cancer-specific therapeutic frameworks will be essential to determine whether IGF-axis modulation can be incorporated effectively into precision oncology for aggressive or radioiodine-refractory disease.

## Conclusion

The IGF signaling network exerts a multifaceted and context-dependent influence on thyroid cancer biology, contributing to tumor progression, aggressive phenotypes, treatment resistance, and dedifferentiation. Dysregulation of IGF ligands, receptors, and IGF-binding proteins profoundly alters proliferative signaling, cellular differentiation, and tumor–microenvironment interactions.

Although early IGF-targeted therapies were limited by pathway redundancy and metabolic toxicity, recent advances – including molecular stratification, high-affinity ligand and receptor inhibitors, nanoparticle-enabled nucleic acid delivery, and IGF-1R-targeted molecular imaging – have renewed translational interest in this pathway, even though clinical validation remains limited. Future biomarker-driven studies focusing on IGF-2/IR-A-dominant tumors may help identify patient subsets most likely to benefit from IGF-axis modulation.

Ultimately, well-designed clinical trials and thyroid cancer-specific therapeutic frameworks will determine how IGF-directed strategies can be effectively incorporated into precision oncology. Collectively, current evidence positions the IGF axis not as a universally dominant target in thyroid cancer but as a biologically important and clinically challenging pathway whose therapeutic relevance will likely depend on biomarker-guided stratification, disease context, and rational combination strategies.

## Declaration of interest

The authors declare that there is no conflict of interest that could be perceived as prejudicing the impartiality of the work reported.

## Author contribution statement

YP conceived and designed the study and wrote, reviewed, and revised the manuscript. SX and HL reviewed and revised the manuscript. XL and YD supervised the study. All authors have read and agreed to the published version of the manuscript.

## Funding

This work was supported by the Tongji Hospital Medical Artificial Intelligence Project (Grant No. AI2025A02), the National Natural Science Foundation of China (Grant No. 82203392), and the Natural Science Foundation of Hubei Province (Grant No. 2026AFB788).

## Ethics statement

This manuscript is a narrative review based solely on previously published studies and publicly available data. The authors did not conduct any new studies involving human participants or animals. Therefore, ethical approval and informed consent were not required for this work.

## Data availability

Data sharing is not applicable to this article, as no datasets were generated or analyzed during the current study.
